# Supplementary material for: Sea Anemone Kunitz-Type Peptides Demonstrate Neuroprotective Activity in the 6-Hydroxydopamine Induced Neurotoxicity Model
Source: Biomedicines. 2021 Mar 10;9(3):283. doi: 10.3390/biomedicines9030283 (PMC8001995; doi:10.3390/biomedicines9030283)
Supplement: Supplementary file 1 [file biomedicines-09-00283-s001.pdf]

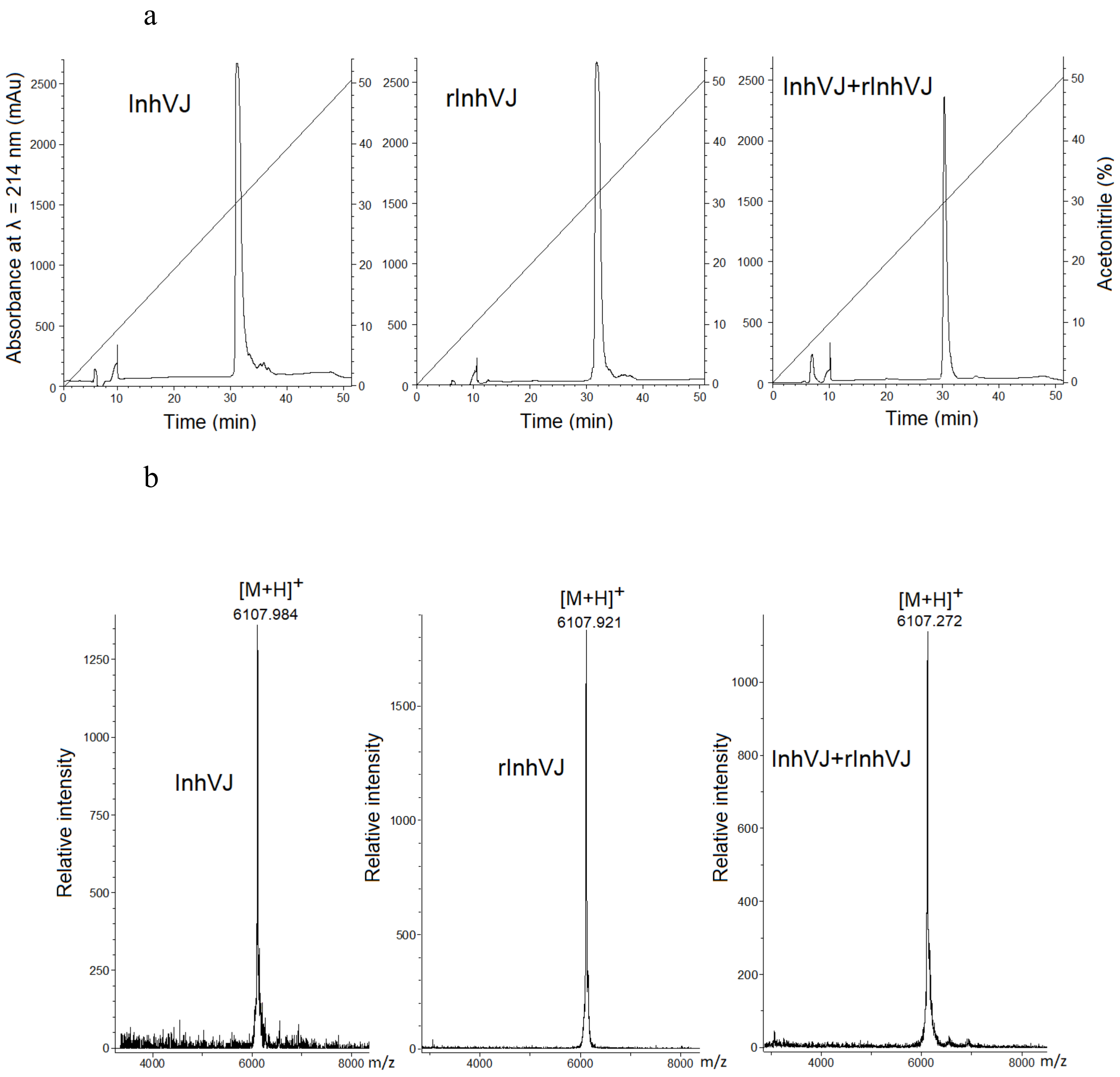

Figure S1 – (a) Elution profile of native IhnVJ, recombinant IhnVJ (rIhnVJ) and their mix on Nucleosil C_{18} column (Sigma Aldrich, USA) in a linear gradient of buffer B (0–50%) at a flow rate of 0.5 ml/min for 50 min. (b) Mass spectra of native InhVJ, recombinant IhnVJ (rIhnVJ) and their mix.

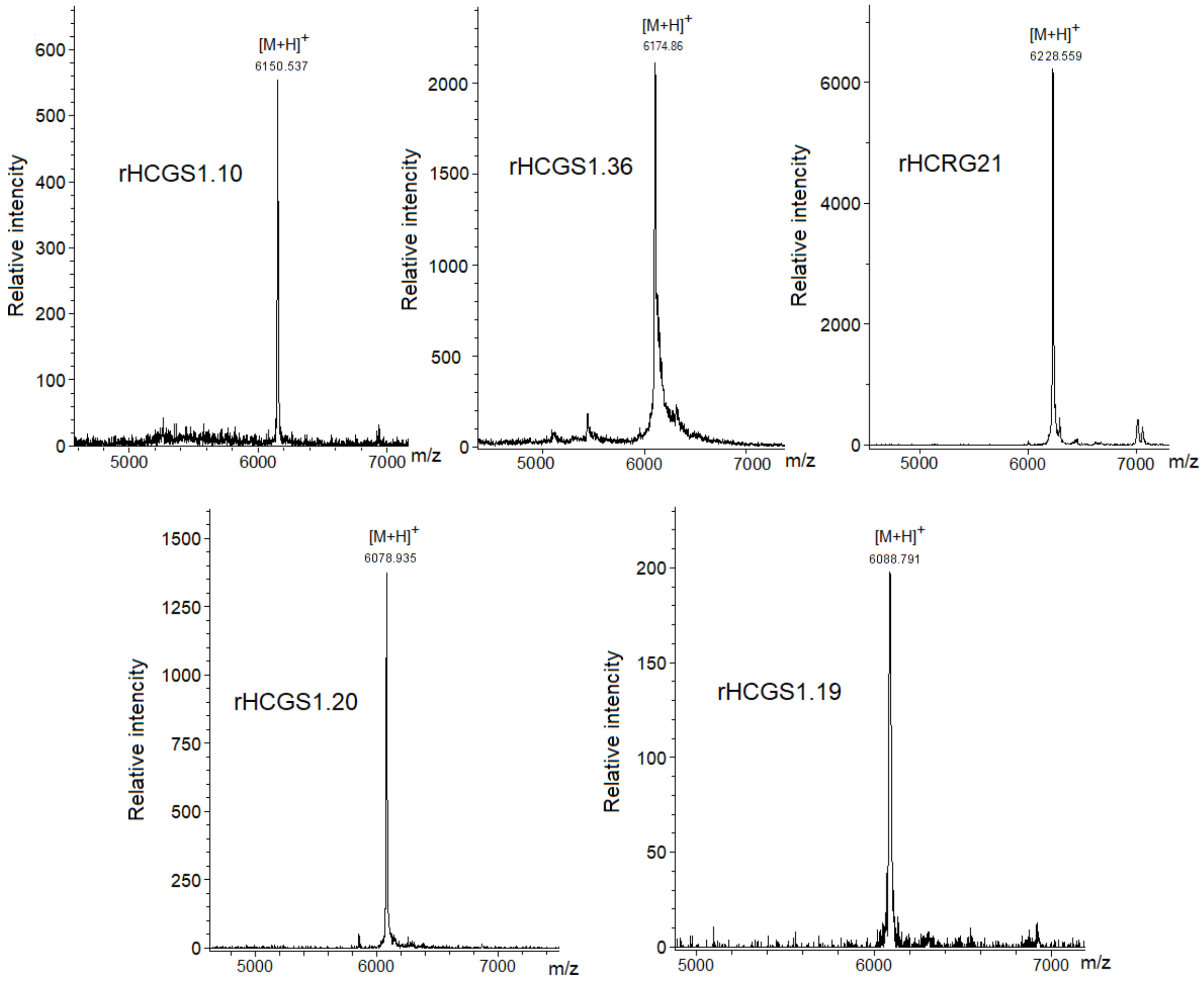

Figure S2 – Mass spectra of recombinant Kunitz-type peptides.
